# Behavioral Neuroscience in the Era of Genomics: Tools and Lessons for Analyzing High-Dimensional Datasets

**DOI:** 10.3390/ijms23073811

**Published:** 2022-03-30

**Authors:** Assa Bentzur, Shahar Alon, Galit Shohat-Ophir

**Affiliations:** 1 The Mina & Everard Goodman Faculty of Life Sciences, Gonda Multidisciplinary Brain Research Center, Institute of Nanotechnology, Bar-Ilan University, Ramat Gan 5290002, Israel; abentzur@gmail.com; 2 The Alexander Kofkin Faculty of Engineering, Gonda Multidisciplinary Brain Research Center, Institute of Nanotechnology and Advanced Materials, Bar-Ilan University, Ramat Gan 5290002, Israel

**Keywords:** behavioral analysis, large datasets, variance

## Abstract

Behavioral neuroscience underwent a technology-driven revolution with the emergence of machine-vision and machine-learning technologies. These technological advances facilitated the generation of high-resolution, high-throughput capture and analysis of complex behaviors. Therefore, behavioral neuroscience is becoming a data-rich field. While behavioral researchers use advanced computational tools to analyze the resulting datasets, the search for robust and standardized analysis tools is still ongoing. At the same time, the field of genomics exploded with a plethora of technologies which enabled the generation of massive datasets. This growth of genomics data drove the emergence of powerful computational approaches to analyze these data. Here, we discuss the composition of a large behavioral dataset, and the differences and similarities between behavioral and genomics data. We then give examples of genomics-related tools that might be of use for behavioral analysis and discuss concepts that might emerge when considering the two fields together.

## 1. Introduction

During the last decade, behavioral neuroscience has gone through a technology-driven revolution with the emergence of machine-vision and machine-learning algorithms. Researchers can now obtain unprecedented high-resolution and high-throughput recordings, tracking and analysis of freely behaving animals. This gives rise to large-scale datasets containing continuous measurements over time of hundreds of behavioral parameters (features) per animal, between pairs of interacting animals, and parameters that characterize emergent group behavior. For example, for many years researchers utilized behavioral features that were based on recorded track paths (using center of mass), and discarded the rest of the video data because storage was constrained by the available hardware. Recent advances in hardware technology can accommodate large data files, and new machine-vision algorithms allow for the tracking of body parts, postures [1,2] and even facial expressions in high resolution [3], giving rise to rich behavioral datasets. For instance, in a behavioral experiment lasting 15 min (with 30 frames per second), up to hundreds of features per animal per frame can be generated, resulting in gigabytes of data per experiment [4,5,6,7,8,9,10,11,12,13,14,15,16,17]. Analyzing such large and complex datasets, researchers use advanced computational approaches (reviewed by [18,19]). However, recent advances in computational approaches in other fields of biology that regularly deal with large datasets, such as genomics, might be beneficial as well. While several fields in biology underwent technological advancement around the same time [20,21,22], standardized protocols and tools for genomic and proteomic analysis developed in a faster manner. This is partially due to the universality of their measured outputs (nucleotides and peptides) and their massive use in many biological systems, unlike tools for behavioral analysis that were developed separately for specific behavioral paradigms in each type of animal.

In this paper, we discuss the possibility of adapting approaches from genomics to behavioral studies. First, we introduce the challenges with analyzing behavioral data, and discuss the similarities and differences between datasets in the fields of genomics and behavior, and then provide examples of approaches from genomics that might fit behavioral datasets.

## 2. Social Behavior Generates High-Dimensional Datasets

Animals execute many motivated behaviors over the course of their lives, with the goal of surviving and reproducing. When these behaviors involve interacting with other members of the same species, they are considered social interactions [23]. In each individual, sensory systems continuously perceive signals from the environment, generating an internal representation of the outside world. This sensory information is integrated with the internal state of the animal, determining how readily sensory stimuli trigger the type and intensity of motor actions [24]. In a social environment, the behavior of individuals is further influenced by and affects the behavior of others, resulting in a highly dynamic environment, where each interaction can change the social context of subsequent interactions, leading to a variety of behavioral outcomes from what seems to be identical starting conditions [7,25,26,27,28,29]. Therefore, social interaction in groups is a dynamic and multi-dimensional phenomenon that is continuously shaped by neuronal processing of external information in each individual. This internal representation determines individual behavioral choices that in turn affect other individuals. The complex nature of this environment imposes conceptual challenges in the quantification and analysis of group behavior.

**Figure 1:**
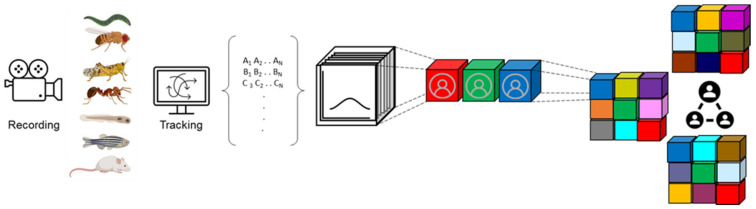
Quantification of behavior generates high-dimensional datasets. Behaviors are recorded over time, the resulting movies are then used to track the position, size, and orientation of all animals in each frame. Tracking data are used to compute various behavioral parameters for each individual per frame, which are then utilized to construct a distribution of behavior between individuals. Finally, interactions between individuals are quantified and are used to generate social network structures (right).

While social interactions in groups give rise to emergent structures that are beyond the sum of the individuals that constitute them, capturing and analyzing such phenomena starts from simple building blocks that are based on the size, orientation, and location of single individuals per frame of the recording (Figure 1). This information is extracted from movies using machine-vision algorithms that track individual animals over time. This seemingly basic information provides a rich repertoire of calculated features such as velocities, angles, changes in velocities, angular velocity, and distances from the center and edge of the arena. Extending these features to account for relative measurements between pairs of animals dramatically increases the number of features per frame, resulting in a rich dataset that can be used to construct social networks and to automatically classify behaviors of single animals and ones that involve several individuals, using machine-learning algorithms (Figure 1). Calculating the duration and frequency of behaviors, number of interactions, length of interactions and types of interactions can give rise to hundreds of features that represent distinct behavioral aspects in each experiment [5,25,30,31] (Figure 1). When individuals interact in a social environment such as a group, the dynamical nature of this environment significantly affects the behavior of individuals that constitute it. One aspect of this is new types of behavioral parameters which are not exhibited when animals are alone, greatly enriching the behavioral dataset (Figure 1). Another aspect of this is that interactions between individuals constantly change their behavior, making them behave differently from how they would when alone, resulting in a somewhat interdependent dataset. Using robust tracking algorithms that maintain the identity of individuals over time, it is also possible to construct social networks that represent an emergent structure from the time-dependent interaction dataset [32,33].

## 3. Both Behavioral and Genomic Datasets Are High-Dimensional

Both genomic and behavioral datasets describe high-dimensional systems, corresponding to collection of molecules in a given cell, repertoire of cells within a tissue, or individuals in a group. A common hallmark shared by all these systems is that they are composed of different organizational levels, each of which possess multiple dimensions. For instance, social groups are a collection of individuals, which are built from tissues that contain different repertoire of cells, containing various molecules, all of which work together to generate composite emergent phenomena. One can describe complex systems by representing their multiple dimensions as variables/features. For instance, a given cell can be described by the repertoire of expressed RNAs and proteins, the combination of which accounts for different cell states, or in the case of a single neuron, the composition of its ion channels determines its possible neuronal output [34]. Neuronal networks can be described by relative ratios of neuronal types and their possible connectivity, whereas behavior can be described by the types and extent of actions depicted by an individual, and social groups can be described by all possible interactions between individuals. In practice, one can use multiple dimensions to describe each organizational layer, giving rise to a high-dimensional space. The conceptual similarity when describing each organizational layer suggests one can implement tools that were originally developed to analyze the complexity of a certain organizational level, such as in single-cell RNA sequencing (scRNAseq) data, to analyze a different level, such as in social group interactions.

## 4. Technological Advances in Other Fields Generate Large Datasets

In the past decade, the fields of genomics and proteomics have led to an explosion of novel insights into the basic building blocks of life and how they are organized. The main drivers in these fields have been technological advancements, which allowed for the generation of massive amounts of molecular data. These technological advancements include for example, scRNAseq in genomics, and improved mass spectrometry analysis in proteomics. Moreover, recently many of these technologies have been applied in situ, generating information about molecules with their 2D or 3D localization [35,36,37,38,39,40,41]. Technological advancements at the molecular level are also evident in functional neuroscience, and include, for example, high-resolution electrophysiological-based and calcium-based recordings of neurons in living, behaving animals [42,43,44,45,46,47]. These advancements increase the resolution and number of molecular or functional elements that are measured in a single sample, leading to an explosion in the amount of data generated from a single experiment [48]. For example, using expansion sequencing [35], ~2 terabytes of image data are generated per one tissue sample (~1 cm by ~1 cm in size).

## 5. Similarities and Differences between Genomics and Behavior Data

The generation of ‘big’ data in the aforementioned fields naturally brings about technical challenges. Both theoretical and computational approaches that fit large datasets are needed, and indeed, much progress has been made in this regard over the last few years [22,48,49,50,51,52,53,54,55,56,57,58,59]. Most of these approaches were developed for genomics, the field that generates the largest amount of data. For this reason, from this point onwards, we will focus on the field of genomics. However, before we introduce some of the computational approaches in genomics/transcriptomics and their possible relevance for behavioral neuroscience, we want to point out a clear difference between the data generated in these two different fields: ‘snapshots’ versus continuous measurement. Data acquisition in transcriptomics is composed of ‘snapshots’ in time, i.e., each dataset represents the expression of all accessible RNA in the sample at a particular time. In most cases, only one ‘snapshot’ is possible per sample; for example, one piece of hippocampal tissue can be thoroughly studied via single-cell sequencing or MERFISH [33], as the tissue is either dissolved or fixed, respectively, in the process. In contrast, in behavioral experiments, data acquisition is continuous, i.e., each dataset represents a value of all behavioral parameters per frame, over the entire duration of the experiment.

The ‘snapshot’ nature of the transcriptomics data leads to questions which focus on the relationship between the examined features (i.e., molecules) within a specific time point. This includes characterizing molecules in cells, defining the cell types present in a sample, and refining the definition of cell types into cell states. These aggregated entities (cell types and cell states) are easier to compare between samples from different ‘snapshots’. In contrast, continuous measurement in behavioral experiments leads to questions which focus on the dynamics, activity and other behavioral aspects over time, and in-group behavior analysis is focused on the emergent social structure over time [60], which can be compared between groups or conditions. Interestingly, new approaches in genomics/transcriptomics allow one to estimate trajectories of genes, cells and circuits from ‘snapshots’ in an attempt to enable a time-dependent analysis (for example, cell-type trajectories [61] and RNA velocity [62,63]).

In addition to the differences with respect to the time domain, two other differences might seem to create a gap between genomics data and behavioral data: the first is the number of measured variables (i.e., number of features), and the second is the dependence between variables. Regarding the number of features, behavioral data are usually characterized by tens to hundreds of features, a number limited by our ability to distinguish unique behavioral characteristics. In contrast, in genomics, thousands or even tens of thousands of features are typically measured, as the dataset scales with the number of measurable genes. As a result, the resulting behavioral datasets might seem to be significantly smaller compared to their genomics equivalents. However, the difference in the number of features is less dramatic when one considers the fact that in behavioral data, each feature is measured in every time frame, multiplying the size of the dataset by the number of frames in the experiment. Regarding the dependence between variables, one might think that measuring many variables in behavioral assays is fundamentally different from measuring gene expression in genomics, because the behavioral variables are more likely to be dependent. However, genes are also highly correlated in their expression and tend to work in concert [64,65,66]. In fact, if the expression of different genes was not strongly correlated, the high dimensionality of the problem would have made it impossible to analyze gene expression in single cells [58]. With the differences and similarities between behavioral and genomics dataset in mind, what kind of approaches can be ‘borrowed’ from genomics? Below are a few examples.

## 6. Dimensionality Reduction

**Figure 2:**
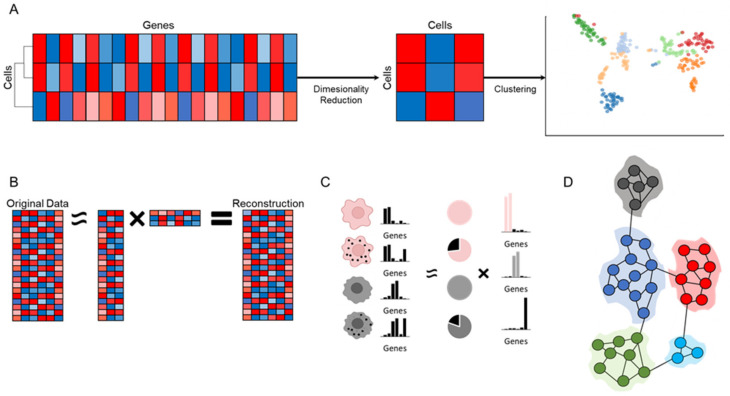
Dimensionality-reduction, clustering and matrix-decomposition approaches used in genomics to analyze expression patterns in high-dimensional datasets. (**A**) Reducing high-dimensional data into lower dimensional space enables clustering of data and visualization of cell types (represented by the different colors). (**B**) PCA, ICA and NMF are all based on matrix decomposition of the original dataset, using a set of describing vectors (reviewed by [67]). In PCA, the describing vectors are the principal components, whereas in ICA these vectors are chosen to maximize their independence. (**C**) In NMF, these vectors are termed gene modules, and can describe either the cell types (pink and gray cells) or cell states, the latter illustrated as a specific gene expressed within the cells (black dots). Image inspired by Kotliar et al. [73]. (**D**) Network Community Detection creates a graph that represents cells (nodes) and cell–cell similarity metrics (edges), which is then used to identify densely connected regions as clusters (reviewed by [74]).

The general idea of dimensionality reduction is to reduce the number of variables without creating a significant loss of information. In genomics, various methods to reduce high-dimensional data into lower dimensional space are routinely used (reviewed by [67]), facilitating easier comparisons between conditions (Figure 2A). Fundamental methods for dimension reduction include principal component analysis (PCA), which finds orthogonal features of maximum variation; independent component analysis (ICA), which finds statistically independent features that best reconstruct the original data (Figure 2B); and nonnegative matrix factorization (NMF), which finds gene modules that combine expression across multiple correlated genes (Figure 2C). In addition to dimension reduction, new methods allow for the visualization of high-dimension data as 3D and even 2D plots that are easier to understand (albeit with reduced ability to perform formal analysis on the resulting plots) (Figure 2A right). These visualization methods include t-distributed stochastic neighbor embedding (t-SNE) [68]; methods based on k-nearest neighbor (KNN) graphs, which can be visualized according to a force-directed layout [69,70]; and the uniform manifold approximation and projection (UMAP) algorithm [71,72]. We note that in behavioral analysis, averaging behavioral variables over time and over individuals is commonly used. This approach can be thought of as a basic type of dimensionality reduction, as a large dataset is replaced with a simpler one in which there is a reduced dependence between the variables and detecting changes between variables is easier. However, adopting formal dimensionality-reduction methods in behavioral data can be informative and can help reveal underlying mechanisms which are not obvious when looking at means.

## 7. Clustering Analysis

In genomics, clustering of data is a powerful tool to gain insights into high-dimensional data [75] (reviewed by [76,77]). This approach allows conversion of the time-dependent expression of thousands of genes into tens of tangible expression ‘profiles’. In many cases, the genes in a given profile share a similar function, elucidating the functional role of the genes involved. Moreover, genes for which no functional information was available have become accessible using this approach. It is easy to see how this approach can be generalized from the time-dependent expression of genes into other time-dependent features (for example in behavioral measurements). In a typical single-cell RNA-sequencing experiment performed nowadays, clustering analysis is performed on the level of the cells. The motivation is to find cells which are similar to one another, therefore converting thousands (or more) of individual cells into tens of ‘types’ of cells (Figure 2A). The higher the number of cell types (or cell states, which are finer scale variations in cells within a cell type), the more homogeneous the cells within each type or state [78,79,80]. The cell groups (i.e., cell types and states) can then be compared to one another or across different experimental conditions in a given cell type. Two popular types of clustering approaches are hierarchical clustering and network community detection [75,77]. Hierarchical clustering works by joining cells iteratively into groups based on similarity metrics. In network community detection, a graph is generated to represent cells (nodes) and cell–cell similarity metrics (edges), and then densely connected regions of nodes are identified as clusters (Figure 2D). Both methods share the need for a cell-to-cell similarity metric. In other words, these methods can detect groups of cells given a similarity metric between them, and therefore defining such a metric becomes the main challenge. This is performed by deciding which genes should be used to define the distance between the cells, a problem which echoes the dimensionality-reduction topic mentioned above.

**Figure 3:**
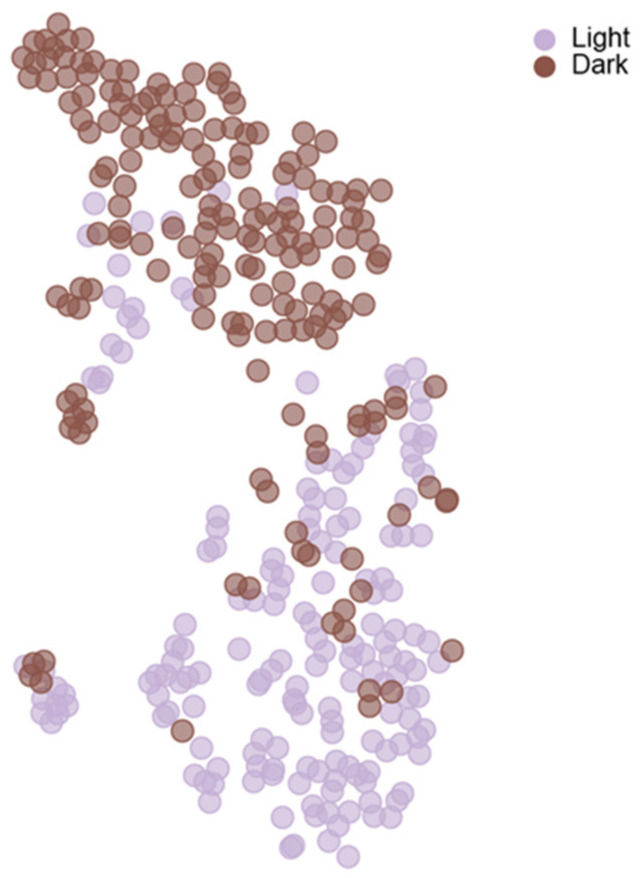
Methods used for visualization of single-cell transcriptomics can be used for behavioral data. t-SNE analysis of all behavioral parameters of individual wild-type flies. Each circle represents one individual. Flies were raised in groups for 3 days prior to testing in a 12 h light/dark cycle and were then tested as a group in FlyBowl arenas, either in light (purple) or in dark (brown) conditions. Behavioral recording and analysis were performed as in Bentzur et al. [25].

Clustering analysis can also be useful to extract meaningful patterns in complex behavioral datasets, where instead of genes/cells, one clusters behavioral parameters and conditions/genetic manipulations [25]. For example, we used hierarchical clustering to compare behavioral signatures of male *Drosophila* flies under various conditions. The behavioral signature of each group contained more than 50 behavioral parameters, including velocities, angles, distance between flies, duration and frequency of behaviors as well as features that describe the formation of social networks. Extracting meaningful patterns from this rich dataset required normalization of the various features followed by their clustering according to conditions and features. This type of analysis allows one to discriminate between conditions or genetic manipulations and to identify groups which exhibit similar patterns. Moreover, clustering of behavioral features can illuminate subsets of clustered parameters that are co-regulated under a certain condition/genotype, providing valuable information about the hallmark of the studied behavior [25]. In addition, one can use visualization algorithms such as t-SNE (broadly used in single-cell RNAseq experiments), to view individuals in 2D across conditions/genotypes, such that every point in the graph represents the behavioral repertoire/signature of an individual. The 2D representation of individuals by types/conditions can be used to test whether populations are composed of individuals that are similar or different from one another, or whether a single population is composed of subgroups, as can be seen in Figure 3.

## 8. Variance as a Tool to Investigate Behavioral Phenomena

When trying to elucidate a biological mechanism, such as how the activity of specific genes affects a specific phenotype, we usually compare the means of experimental groups to controls, with the hope to record low variability between repeats for high statistical significance between the means of tested groups [81]. Behavioral data are notoriously variable, mainly because of inter-individual differences in voluntary actions and high sensitivity to even mild environmental changes, both of which pose a challenge to reproducing experimental results. Therefore, reducing behavioral variance is desirable in order to increase our ability to resolve the mean of an effect, mostly by increasing the number of repeats per experiment and by better control of test conditions [81].

Nevertheless, studying behavioral variance can have an added value, as differences between populations can exist in the distribution of the data (variance), without affecting averages [82]. Indeed, there are interesting examples for how variance is an additional and valuable feature to describe biological phenomena. For example, in the field of ecology, increased variance of certain parameters in a group is known to increase survivability of the group in a changing environment, a phenomenon known as bet hedging [83]. This means that actions taken by certain individuals are sub-optimal for their survival and reproduction in a constant environment but could be beneficial in an unpredictable environment. A classic example of this is the ratio between germinated and ungerminated plant seeds in a given year [83].

Applying this principle to social environments, which are inherently competitive and unpredictable, suggests that behavioral differences between individuals in a group can contribute to its overall success [84]. Interestingly, even in highly synchronized collective behaviors of schooling fish and swarming locusts, which are expected to have low variance between individuals, some level of interindividual variance is still required. Knebel et al. show that in locusts, inter-individual behavioral differences drive differences in inter-group differences, and that this can explain certain attributes of swarm formation [85]. Jolles et al. show that in fish, heterogeneous groups can form collective behaviors based on consistent inter-individual differences [10].

Social interactions between members in a group are a driving force for variance between individuals, since each individual develops its own unique interaction sequence (i.e., with which group member, in what order, type of interaction, and its duration). Even assuming similar starting states for all individuals, the “trajectory” of each individual is actively determined by its interaction sequence, thus creating differences between members of the group [25]. Any given behavioral feature is represented by a series of values over time, per individual. One can extract from this raw data a distribution of values over time between all individuals (inter-individual variance) in a group and between groups (inter-group variance). These measures are useful as additional dimensions to the behavioral dataset, and as a tool to understand the contribution of prior experience, group composition or other biological aspects to social group dynamics. For instance, groups composed of socially raised *Drosophila* males exhibit high inter-individual and high inter-group variation in various behavioral features compared to groups composed of male flies that were raised in social isolation [25]. This implies that social enrichment facilitates behavioral variability between groups, such that the range of possible group dynamics exhibited by different groups is larger when they are composed of individuals with prior social experience.

## 9. The Concept of Individuality and Group Identity

A well-documented phenomenon in behavioral ecology is that individuals exhibit certain characteristics that are maintained over time. These can include the amount of attraction or aversion to others, relative activity levels and other attributes that are maintained by specific individuals over the duration of the experiment, and are considered as persistent inter-individual differences [86,87,88,89]. For example, Stern et al. showed that in c. elegans, even isogenic animals in a similar environment can have consistent behavioral biases that differ from the population average, and that this is regulated by neuromodulatory mechanisms [86], suggesting that persistent inter-individual differences are a result of specific biological mechanisms. Recent advances in computational neuroscience for analyzing high-dimensional behavioral data allow us to resolve inter-individual differences and even specific personalities, which can account for part of the overall variance in a population [90]. It will be interesting to see whether it is possible to connect these two aspects by attempting to correlate certain personality types with differences in the same mechanisms that underlie consistent behavioral biases.

One can extend the idea of individuality to social groups, where each group exhibits consistent features maintained over time that emerge from interactions between members of the group, suggestive of distinct group identities. For instance, when analyzing variance between groups composed of *Drosophila* flies that were raised in isolation or in groups, socially experienced flies exhibit higher inter-group variance, suggesting that each group developed emergent characteristics that make it distinguishable from other groups with seemingly identical individuals [25]. A recent study investigated the factors that shape and maintain variation in group structure in *Drosophila melanogaster*. Focusing on the positions of individuals within the network as a feature that shows variability between groups, they discovered that genotypes of the individuals comprising the group, their prior experience and environmental conditions contribute to inter-group variation [91]. Altogether, behavioral variation in group structure shares conceptual similarities with bet hedging. This raises interesting questions about the molecular and neuronal mechanisms that maintain behavioral persistence of both individuals and groups: does inter-group variance increase the survivability of groups in a changing environment, what is the effect of group size on survivability, and which neuromodulatory mechanisms control the magnitude of variance between groups?

## 10. Variance and Individuality in Genomics

An interesting case study for the possible learning opportunities from genomics in social behavior and vice versa is gene expression variance. Variance in expression per gene is tightly regulated in the level of tissues and organisms [92,93]. Changes in this regulation due to genetic mutations can create changes in morphological and physiological traits and give rise to disease states [92,93]. Single-cell genomics allows us to measure the variance in gene expression between individual cells. However, explaining the source of this measured variance was elusive until recently. While it is known that stochasticity plays a major role in gene expression variance at the single-cell level, not enough was known about other possible sources of the measured variance. With the emergence of the field of spatially resolved transcriptomics, it is now possible to physically position the individual cells in tissues. This allows us to break down the variance in gene expression into additional measurable sources. In other words, how much of the variance in gene expression can be explained by cell type? How much of the remaining variance is explained by physical location in the tissue, or by cell–cell interactions? [94,95,96,97,98,99]. The analytic tools used to quantify the contributing factors to gene expression variance in single cells can potentially be of use for quantifying variance in the measurements of behavior in individuals in social settings [94,95,96,97,98,99]. The analogy here is that individuals can be akin to cells, leading to questions about what part of the behavioral variance is explained by certain parameters, such as distance between individuals, their interactions, etc. This analogy might also be useful in the other direction as well: is it time to start thinking about ‘individuality’ in cells? The prevailing way of thinking about cells is that they are characterized by their cell type; however, we are getting better and better at measuring cell states, and connecting them to physical factors, such as proximity to other cells, location in tissue, exposure to environmental factors such as hypoxia, and so on. These differences between individual cells of the same type, which are manifested in gene expression, can be persisted over time (which is not trivial to measure) or over space, as outlined above, and therefore can contribute to the concept of ‘individuality’ in cells. Evidence for this concept can be seen in a recent study demonstrating that variation in gene expression between cells of isogenic melanoma cells, that were previously considered as noise, are in fact persistent differences that are maintained over several cell divisions [100]. Lastly, Phillips et al. demonstrated that daughter cells in embryonic cell lineages maintain similar patterns of gene expression to their mother cells, providing a mechanistic explanation for the formation of inter-cell variation in gene expression patterns within tissues [101].

## 11. Future Perspective and Challenges

A hallmark of genomics research over the last two decades is data accessibility and standardization of analysis, an issue which is still lacking in the field of behavioral neuroscience research [102]. Ideally, this means that all raw data from each experiment are deposited in a way which is accessible to everyone and can be processed using standard tools which keep improving using community involvement. Software engineers are now routinely involved in the storage, processing and streamlining analysis of genomics data. As a result, constantly updated software is available for the community, for example the Seurat R toolkit [103,104] and Scanpy [105] for normalization, scaling, transformation and processing. Moreover, analytic tools allow for direct comparison between the analysis of multiple datasets which were generated by the same lab at different times, different labs, or even different modalities of measurements, without the need to re-analyze the raw data. Two examples of such an analysis are canonical correlation analysis (CCA), which aims to identify a set of variables that are maximally correlated between two datasets, and mutual nearest neighbors (MNNs), which detects variables mutually closest to each other across datasets [104].

Currently, all genomic-related studies are required to deposit the raw data at public archives, such as the Gene Expression Omnibus (GEO) database. This, in combination with standardized methods for sample preparation and analysis, facilitates further investigation of the original data by other research groups, and even allows for comparison between datasets from different sources. This is far from being the case for behavior datasets, due to various reasons. While the building blocks in genomics are strings of nucleic acids, it is hard to find a uniform basic element common to all behavioral measurements. It is even challenging to unify all features from a single experiment that contains velocities (mm/sec), angle (rad), distances (mm), behaviors (% of frames) and durations of behaviors (sec), requiring normalization of all features using approaches such as Z-Scoring. Many labs do not even compare between experiments that were conducted on different days due to batch effects of day-to-day differences in behavior that result from mild variation in environmental conditions, nevermind of comparing animals with different genetic backgrounds. Adding to that is the use of various behavioral paradigms, arenas and lighting conditions that require adjusting tracking algorithms to each experimental setup. Lastly, behavioral neuroscience investigates a variety of animals, with different anatomical shapes, sizes and complex behaviors.

Although there are many challenges that impede standardization of behavioral data, some machine-vision algorithms such as CTRX [106] and DeepLabCut [107] are capable of tracking various organisms, providing hope that it will be possible at some point to compare between experiments and maybe model organisms using several basic features. While acquisition is achieved using different methods, we believe that it is possible to find universal parameters that can facilitate the standardization of the data. Firstly, these include general information about the experimental setup: type of animal, genotype, sex, age, and time of day is necessary. Secondly, technical specifications: illumination, frame rate, arena size and shape, temperature and humidity, number of animals and other parameters are crucial to create a comparable dataset. Lastly, parameters of the animal’s coordinates, dimensions of its fitted shape and orientation per frame for the duration of the experiment. Depositing this raw data will facilitate analysis by other researchers, which also raises questions of how to optimize data storage when datasets are quickly growing. Maybe the most promising field in this respect is the study of social networks, which uses a set of features that describe individuals within a network and compares between different networks under various conditions. A step towards this direction was recently achieved in a study by Jezovit et al. [108], who compared the results of several studies that focused on social networks in *Drosophila melanogaster*. Although each study used a different approach to calculate network features, the authors documented similar effects of social isolation on network structure across all studies. Several studies attempted to compare social network structures between different organisms [109,110], suggesting that there are similarities in structures of groups from different species, although these datasets were generated using different techniques [111]. This further emphasizes the need to use a standardized set of basic behavioral features when depositing datasets. An in-depth discussion of the optimal set of features for this is instrumental in advancing the field of behavioral neuroscience.

To conclude, behavioral neuroscience is shifting towards large data. This poses several challenges and opportunities for advancing our understanding of phenomena which were once hard to analyze, such as behavioral variance. To advance the field, we can adopt approaches from other fields such as genomics and decide on protocols for best-practice dataset deposition, which will enable the development of analysis toolkits and other community resources.
